# OLFML2B promotes hepatocellular carcinoma malignancy via the PI3K/AKT-EMT axis and correlates with an immunosuppressive tumor microenvironment

**DOI:** 10.3389/fimmu.2026.1779625

**Published:** 2026-08-10

**Authors:** Xiaoling Liu, Yonghong Wang, Jingjing Bai, Cunliang Deng, Changfeng Sun

**Affiliations:** 1 Department of Infectious Diseases, Affiliated Hospital, Southwest Medical University, Luzhou, China; 2 Laboratory of Infection and Immunity, The Affiliated Hospital, Southwest Medical University, Luzhou, China; 3 Department of Tuberculosis, Affiliated Hospital, Southwest Medical University, Luzhou, China

**Keywords:** hepatocellular carcinoma, OLFML2B, PI3K/Akt pathway, epithelial-mesenchymal transition, proliferation, metastasis, drug sensitivity

## Abstract

**Introduction:**

Hepatocellular carcinoma (HCC) is a leading cause of global cancer-related mortality, highlighting the need for novel biomarkers and therapeutic targets.

**Methods:**

The role of Olfactomedin-like 2B (OLFML2B) in HCC was investigated through multi-database analyses (The Cancer Genome Atlas, International Cancer Genome Consortium, Gene Expression Omnibus) and experimental validation.

**Results:**

OLFML2B was significantly upregulated in HCC tissues, correlated with poor overall and disease-specific survival, clinicopathological features (tumor grade, stage, age, gender), and robust diagnostic performance (AUC > 0.7 across 14/15 datasets). Transcriptomic and single-cell analyses further revealed that high OLFML2B expression was associated with an immunosuppressive tumor microenvironment, characterized by increased infiltration of M2 macrophages, cancer-associated fibroblasts (CAFs), and regulatory T cells (Tregs), as well as reduced abundance of cytotoxic T cells and NK cells. Knockdown of OLFML2B suppressed malignant phenotypes, including cell proliferation, migration, invasion, and angiogenesis, attenuated PI3K/AKT-EMT signaling, and enhanced sensitivity to sorafenib, cabozantinib, and regorafenib in Huh7 and HepG2 cells. Additionally, OLFML2B knockdown suppressed tumor growth and metastasis in zebrafish xenografts.

**Discussion:**

Collectively, these findings indicate that OLFML2B is required for HCC progression and represents a prognostic biomarker and potential therapeutic target.

## 1 Introduction

Olfactomedin-like 2B (OLFML2B), also known as photomedin-2, is a secreted glycoprotein that belongs to the subfamily IV of the olfactomedin (OLF) family, with predominant expression in the adult retina (1). All members of the olfactomedin domain-containing protein family possess a specific region consisting of 250 amino acids at their carboxyl (C)-terminus, which is defined as the olfactomedin domain (2–6). OLFML2B contains a unique serine/threonine-rich region, a feature absent in other members of the OLF family (7, 8). Functionally, this unique region enables OLFML2B to specifically bind to chondroitin sulfate-E or heparin in the extracellular matrix, providing a structural basis for its involvement in extracellular matrix-related biological processes, such as cell adhesion, signal transduction, and tissue remodeling (8).

Accumulating evidence implicates OLFML2B as a critical regulator of the progression of multiple malignancies. For instance, in the context of gastric cancer, bladder cancer, and renal cell carcinoma, OLFML2B overexpression correlates with aggressive tumor phenotypes. It serves as a potential diagnostic and prognostic biomarker (9–13). Furthermore, studies have demonstrated that abnormal expression of OLFML2B is closely associated with cancer-associated fibroblasts (CAFs) and epithelial-mesenchymal transition (EMT) (13–15), and that it can reshape the tumor immune microenvironment and induce macrophage polarization toward the M2 phenotype (16).

In 2022, primary liver cancer was the sixth most common cancer globally and the third leading cause of cancer deaths, with hepatocellular carcinoma (HCC) as its predominant subtype. Aberrant expression of OLFML2B has been detected in HCC tissues, and this is strongly associated with poor overall survival and disease-free survival in HCC patients (17). However, the role of OLFML2B in HCC remains unclear.

To further clarify the clinical significance of OLFML2B in HCC, we conducted a comprehensive analysis of the correlations between OLFML2B expression and key clinicopathological features, as well as the immune microenvironment, using data from The Cancer Genome Atlas (TCGA), International Cancer Genome Consortium (ICGC), and Gene Expression Omnibus (GEO) public databases. Our findings revealed that elevated OLFML2B expression strongly correlates with poorer overall survival (OS) in HCC patients and is significantly associated with M2 macrophage infiltration. *In vitro* studies further demonstrated that OLFML2B knockdown markedly inhibits HCC cell proliferation, metastasis, microtubule formation, and EMT. Collectively, this study comprehensively explores the functional role and molecular mechanisms of OLFML2B in HCC progression, aiming to identify it as a novel prognostic biomarker and potential therapeutic target for clinical HCC management.

## 2 Materials and methods

### 2.1 Data acquired

Gene expression and clinical data for patients with HCC from The Cancer Genome Atlas (TCGA) were retrieved from the UCSC Xena browser (https://xenabrowser.net/). The ICGC-LIRI-JP dataset and corresponding clinical information were acquired from the International Cancer Genome Consortium (ICGC) portal (https://dcc.icgc.org/). The dataset GSE22058, GSE25097, GSE36376, GSE14520, GSE54236, GSE63898, GSE64041, GSE76427, GSE60502, GSE102079, GSE164760, GSE6764, GSE135631, GSE202853, GSE46444, GSE140228, GSE125449, GSE149614, and GSE156625 were downloaded from the Gene Expression Omnibus (GEO, https://www.ncbi.nlm.nih.gov/gds). The key information of these datasets was detailed in **Supplementary Table 1**.

### 2.2 Survival analysis

Patients with complete survival information and a survival time of > 30 days were included in the analysis. Kaplan-Meier survival curves were generated using the “survival” and “survminer” packages in R, and survival differences between groups were compared with the log-rank test.

### 2.3 Enrichment analysis

Gene Set Enrichment Analysis (GSEA) (18): GSEA was conducted using the “ClusterProfiler” package in R with predefined gene sets from the MSigDB website (https://www.gsea-msigdb.org/gsea/index.jsp). Gene sets were considered significantly enriched when |Normalized Enrichment Score (NES)| > 1, False Discovery Rate (FDR) < 0.25, and adjusted p-value < 0.05.

Generalized Range Swap Algorithm (GRSA) (19): GRSA was performed via the “ReporterScore” package in R. First, the gene expression matrix was integrated with differential analysis metrics (e.g., p-values, log2 fold change [log2FC]). Next, “Reporter Scores” for individual genes and enrichment scores for predefined gene sets (e.g., KEGG hallmark pathways) were calculated to quantify associations between genes/pathways and HCC. Finally, significantly enriched pathways (adjusted p-value < 0.05) were visualized.

### 2.4 Cell culture

The human liver cancer cell lines HepG2 and Hep3B were purchased from Guangzhou Alexan Biotech Co., Ltd. and the Shanghai Cell Bank of the Chinese Academy of Sciences, respectively. The human HCC cell line Huh7 and the embryonic kidney cell line 293T were purchased from Guangzhou Kefan Biotechnology Co., Ltd. Immortalized human umbilical vein endothelial cells (HUVECs) were purchased from Guangzhou Science and Technology Xiaofan Company. HepG2, Huh7, and 293T were cultured in high-glucose DMEM medium supplemented with 10% fetal bovine serum (FBS). HUVECs were maintained in HUVEC-specific complete medium (Procell, Wuhan, Hubei, China). Hep3B cells were cultured in Minimum Essential Medium (MEM) supplemented with sodium pyruvate and non-essential amino acids. All cells were incubated in a humidified atmosphere with 5% CO_2_ at 37°C, and their authenticity was verified by short tandem repeat (STR) profiling. Transient transfection was conducted using Lipofectamine™ 3000 reagent (Invitrogen, USA) following the manufacturer’s instructions. Penicillin-streptomycin (100×) was added to the medium as needed.

### 2.5 Acquisition of stable transfection cell lines

Log-phase 293T cells (1×10^6^ cells/60-mm dish) were transfected with PLKO.1-OLFML2B shRNA/PLKO.1-NC-shRNA (negative control), psPAX2, and pMD2.G plasmids (4:3:1 ratio) using polyethyleneimine (PEI). Transfected cells were maintained in DMEM supplemented with 2.5% FBS for 72 h, with culture supernatants collected every 24 h. Pooled supernatants were filtered (0.45 μm), mixed with PEG8000 (5%-8% (w/v)) and 0.3 M NaCl, and incubated overnight at 4 °C. After centrifugation (4, 000×g, 30 min, 4 °C), viral pellets were resuspended in pre-chilled sterile PBS (1/100-1/200 of original volume), aliquoted, and stored at -80 °C (avoiding freeze-thaw cycles). HCC cells were infected with the virus in the presence of Polybrene, and stably transfectants were selected with 2 μg/mL puromycin for 2 weeks.

### 2.6 Real-time quantitative PCR

Total RNA was extracted using the total RNA extraction kit (Tiangen, Beijing, China), and 500 ng RNA was reverse-transcribed into complementary DNA (cDNA) using an RNA reverse transcription kit (Takara, Beijing, China). qRT-PCR was performed according to the instructions of a qRT-PCR reaction kit (Takara, Beijing, China), and the relative gene expression was calculated using the 2-ΔΔCT method. The primers used in this study are shown in **Supplementary Table 2**.

### 2.7 Western blot

Proteins were extracted using a membrane and cytosol protein extraction kit (Beyotime, Shanghai, China), and concentrations were determined using a BCA protein assay kit (Biomed, Beijing, China). Equal amounts of proteins were separated by sodium dodecyl sulfate-polyacrylamide gel electrophoresis (SDS-PAGE) and transferred to polyvinylidene difluoride (PVDF) membranes, blocked with protein-free rapid blocking solution (20 min, room temperature), and incubated with primary antibodies overnight at 4°C. After washing, membranes were incubated with secondary antibodies for 1 hour at room temperature. Bands were visualized using an ultra-sensitive ECL chemiluminescence substrate and imaged via a chemiluminescence system. Band intensities were quantified using ImageJ software. Antibody details are in **Supplementary Table 3**.

### 2.8 RNA sequencing

Total RNA was extracted from hepatic cells using TRIzol reagent, and purity/integrity were assessed using a Nanodrop and an Agilent 2100. mRNA was enriched with oligo(dT) beads, fragmented, and reverse-transcribed into cDNA. Libraries were constructed, quantified, and sequenced on an Illumina NovaSeq 6000 platform. Raw reads were filtered; clean reads were aligned to the reference genome for gene expression quantification.

### 2.9 Immunohistochemistry

Paraffin-embedded tissue sections (4 μm) were deparaffinized in xylene, rehydrated through graded ethanol (100%, 95%, 85%, 75%), and subjected to antigen retrieval by boiling in citrate buffer (pH 6.0) for 15 min. Endogenous peroxidase activity was quenched with 3% H_2_O_2_ for 10 min, followed by blocking with 5% bovine serum albumin for 30 min at room temperature. Sections were incubated overnight at 4 °C with rabbit anti-OLFML2B primary antibody (1:200). After washing with PBS, HRP-conjugated goat anti-rabbit IgG secondary antibody was applied for 30 min at room temperature. Immunoreactivity was visualized with DAB, and sections were counterstained with hematoxylin, dehydrated, and mounted. Negative controls were prepared by substituting the primary antibody with PBS. Staining was evaluated independently by two blinded pathologists.

### 2.10 Cell counting kit-8 assay

At 24 h post-transfection, HCC cells (3×10³ cells/well, 5 replicates/group) were seeded in 96-well plates. CCK-8 reagent (10 μL, GLPBIO, USA) was added at 0, 24, 48, 72, and 96 h, followed by 3 h incubation. Absorbance at 450 nm was measured with a microplate reader.

### 2.11 Transwell assay

At 24 h post-transfection, 100 μL serum-free cell suspension (5×10^5^ cells/mL) was seeded into Transwell chambers (Matrigel-precoated for invasion assay, 50 μL 3 mg/mL, BD, Franklin Lakes, NJ, USA). Chambers were placed in 24-well plates with 500 μL of complete DMEM. After 48 h, cells were fixed (4% paraformaldehyde for 30 min), stained (0.1% crystal violet for 20 min), and non-migrated/invaded cells were removed. Five random fields of view were imaged under a microscope (100× magnification) for cell counting.

### 2.12 Scratch assay

HCC cells (4×10^5^ cells/well) were seeded in 12-well plates, cultured overnight, and transiently transfected. At 100% confluency, a straight scratch was made with a sterile 200 μL pipette tip. The cells were then washed three times with PBS to remove detached cells. Images of the scratch area were captured under an inverted microscope at 0 and 48 h (40× magnification), and the healing rate was quantified using ImageJ.

### 2.13 Cell cycle assay

Cells were collected at 72 h post-transfection, washed three times with pre-chilled PBS, and fixed with 70% ethanol overnight. After washing three times with PBS, cells were stained with 500 μL of propidium iodide (PI) staining solution containing RNase (BD, USA) for 15 min in the dark. Flow cytometry was performed, and the cell cycle distribution was analyzed using ModFit software.

### 2.14 Apoptosis assay

Cell apoptosis was assessed using the Annexin V-7-AAD/PI Double Staining Kit (BD Biosciences, Cat. No. 556547). HCC cells (2×10^5^ cells/well) were seeded in 12-well plates and cultured for 72 h. Cells were harvested by trypsin-EDTA digestion, washed twice with cold PBS, and resuspended in 1×binding buffer (1×10^6^ cells/mL). A 100 μL aliquot of the cell suspension was stained with 5 μL of Annexin V-FITC and 5 μL of PI for 15min in the dark. Subsequently, 400 μL of 1× binding buffer was added, and the apoptosis rate was analyzed within 1 h using a BD FACSCanto™ II flow cytometer.

### 2.15 Angiogenesis assay

96-well plates were pre-coated with Matrigel (50 μL, 6.5 mg/mL, 37°C, 1 h). Starved HUVECs (24 h) were mixed with transfected HCC cell supernatant, and 3×10^4^ cells/well (100 μL) were seeded. Images were captured at 0, 6, and 12 h (100× magnification). Vascular junctions and total length were analyzed via ImageJ.

### 2.16 Zebrafish xenograft model experiment

CZ63 (is5Tg, Tg(kdrl:mCherry)) is a transgenic zebrafish line in which endothelial cells specifically express red fluorescent protein, with the kdrl gene promoter driving the expression of mCherry. CZ63 (is5Tg, Tg(kdrl:mCherry)) zebrafish obtained from the Zebrafish Platform of Southwest Medical University were maintained at 28.5 °C (14: 10 h light: dark cycle). To maintain transparency, zebrafish were cultured in Danieau’s Buffer supplemented with 0.03% 1-phenyl-2-thiourea (PTU) starting from 6–24 hours post-fertilization (hpf). The green fluorescent protein (GFP)-labeled HepG2 cells were trypsinized, washed with phosphate-buffered saline (PBS), and resuspended in serum-free medium at a density of 1×10^7^ cells/mL. Approximately 400 cells were microinjected into the yolk sac of 48 hpf embryos. Media was supplemented with 100 μg/mL penicillin/streptomycin post-injection, with daily half replacement and survival monitoring. Cell proliferation/metastasis was observed at 0, 2, and 4 days post-injection (dpi) via Olympus SZX16 stereo fluorescence microscope (168 mg/L tricaine methanesulfonate (MS-222) anesthesia). At 4 dpi, larvae were euthanized via 1000 mg/L MS-222 (30 min), followed by physical decapitation as a definitive secondary measure to ensure complete and irreversible death.

### 2.17 Statistical analysis

Data are expressed as mean ± standard deviation (mean ± SD). Statistical analyses were conducted using SPSS 23.0 software (IBM, Armonk, New York, USA) or GraphPad Prism 10.0 software (GraphPad Software, Inc., San Diego, California, USA). Intergroup comparisons were performed using t-tests or One-way analysis of variance (ANOVA). Cox regression analysis was implemented with the “glmnet” package in R 4.3 (R Foundation for Statistical Computing, Vienna, Austria). All experiments were independently repeated three times. Statistical significance was defined as p < 0.05.

## 3 Results

### 3.1 Highly expressed OLFML2B as a diagnostic marker for HCC

**Figure 1:**
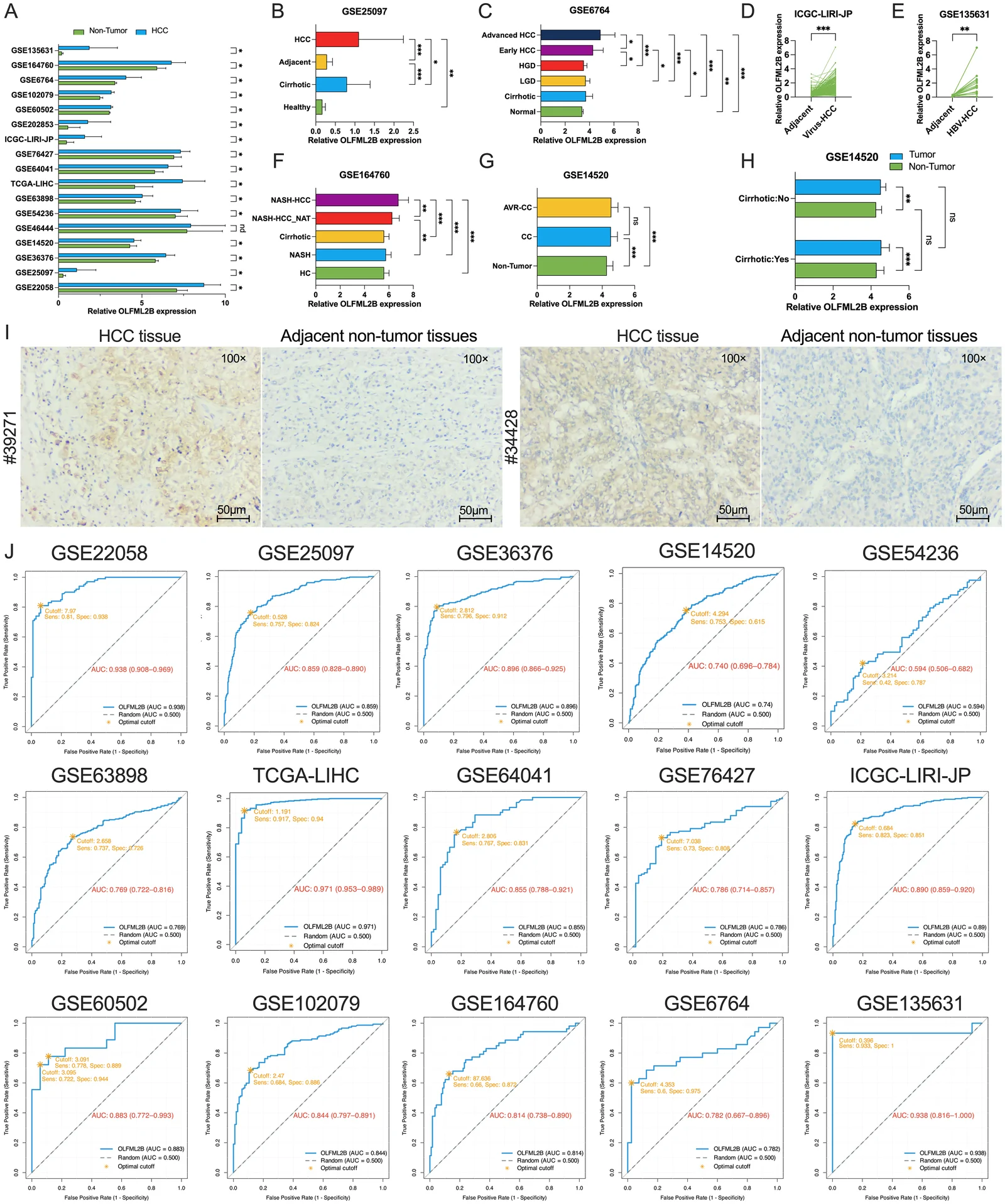
Expression patterns and diagnostic potential of OLFML2B in HCC. **(A)** Expression patterns of OLFML2B in 17 HCC-related datasets. **(B)** OLFML2B expression profiles in HCC, adjacent non-tumor, cirrhotic, and healthy liver tissues of GSE25097. **(C)** OLFML2B expression profiles in advanced HCC, early HCC, HGD, LGD, cirrhotic, and normal liver tissues of GSE6764. **(D)** Expression patterns of OLFML2B in Virus-associated HCC and paired adjacent non-tumor tissues in the ICGC-LIRI-JP dataset. **(E)** Expression patterns of OLFML2B in HBV-associated HCC and paired adjacent non-tumor tissues in the GSE13563 dataset. **(F)** Expression patterns of OLFML2B in NASH-associated HCC and different types of non-tumor tissues in the GSE164760 dataset. **(G)** Expression patterns of OLFML2B in HCC patients with chronic carriers (CC) or active viral replication chronic carriers (AVR-CC) in the GSE14520 dataset. **(H)** Expression patterns of OLFML2B in HCC patients with or without cirrhosis in the GSE14520 dataset. **(I)** Representative immunohistochemical (IHC) staining of OLFML2B in paired HCC and adjacent non-tumor tissues from two patients (#39271 and #34428). Stronger brown cytoplasmic staining indicates higher OLFML2B protein levels in HCC tissues compared to adjacent non-tumor tissues. Scale bar, 50 μm; original magnification, 100×. **(J)** Diagnostic power of OLFML2B across 15 HCC-related datasets. HCC, hepatocellular carcinoma; HGD, high-grade dysplasia; LGD, low-grade dysplasia; NASH, non-alcoholic steatohepatitis; HC, healthy control; TCGA, the Cancer Genome Atlas; LIHC, liver hepatocellular carcinoma. *p< 0.05, **p< 0.01, ***p< 0.001; nd, not detected. HCC, hepatocellular carcinoma; HBV, Hepatitis B virus.

To elucidate OLFML2B expression patterns in HCC, 17 HCC-related datasets were compiled from the TCGA, ICGC, and GEO databases. The results demonstrated that OLFML2B expression was significantly upregulated in HCC tissues compared to non-HCC tissues across the majority of included datasets, with GSE46444 as the sole exception (**Figure 1A**), which not only verifies that OLFML2B is commonly highly expressed in HCC but also offers evidence for subsequent investigations into its functional role and clinical relevance in HCC progression.

Given that HCC develops via a pathological cascade involving liver fibrosis and cirrhosis, OLFML2B expression was compared across liver tissue types using GSE25097 and GSE6764. OLFML2B was significantly overexpressed in HCC tissues relative to cirrhotic or normal liver tissues, while no notable disparity in its expression was detected between cirrhotic and normal liver tissues (**Figures 1B, C**). Notably, OLFML2B was even more highly expressed in early-stage HCC than in high-grade/low-grade dysplastic (HGD/LGD) tissues, and HGD/LGD tissues showed no significant difference from cirrhotic/normal tissues (**Figure 1C**), suggesting its high expression may be specific to HCC.

To investigate OLFML2B expression alterations in HCC driven by two primary etiologies, viral infection (predominantly hepatitis B virus, HBV) and non-alcoholic steatohepatitis (NASH), we compared the expression of OLFML2B in the ICGC-LIRI-JP (virus-associated HCC), GSE135631 (HBV-associated HCC), and GSE164760 (NASH-associated HCC) datasets. The results demonstrated that OLFML2B expression was significantly upregulated in HCC tissues compared with non-tumor tissues, regardless of viral infection or NASH status (**Figures 1D–F**).

To further explore the effects of HBV replication status and cirrhosis on OLFML2B expression, the GSE14520 dataset was analyzed. There was no significant difference in OLFML2B expression between HCC tissues derived from patients with chronic carriers (CC) and active viral replication chronic carriers (AVR-CC), yet expression in both CC- and AVR-CC-derived HCC tissues was markedly higher than in non-tumor tissues (**Figure 1G**). Additionally, regardless of the presence of cirrhosis, OLFML2B expression in HCC tissues was higher than that in non-tumor tissues, with no significant statistical difference observed between HCC tissues from patients with or without cirrhosis (**Figure 1H**).

In addition, to compare OLFML2B protein expression between HCC and adjacent non-tumor tissues, we performed immunohistochemical staining. The results revealed that the OLFML2B protein was markedly upregulated in HCC tissues compared with the corresponding adjacent normal tissues (**Figure 1I**; **Supplementary Figure 1**).

These results indicate that OLFML2B is overexpressed in HCC and shows promising diagnostic performance. To evaluate the diagnostic efficacy of OLFML2B for HCC, we plotted receiver operating characteristic (ROC) curves for 15 independent datasets. After stratification by area under the curve (AUC) values, the results showed that 3 datasets (GSE22058, GSE135631, TCGA-LIHC) had an AUC > 0.9; 7 datasets (e.g., GSE25097 and ICGC-LIRI-JP) had an AUC > 0.8; and 4 datasets (e.g., GSE14520) had an AUC > 0.7. Only GSE54236 had an AUC below 0.7 (**Figure 1J**). Collectively, these findings confirm that OLFML2B has reliable diagnostic efficacy for differentiating HCC tissues from non-tumor liver tissues.

### 3.2 OLFML2B overexpression correlates with unfavorable prognosis in HCC patients

**Figure 2:**
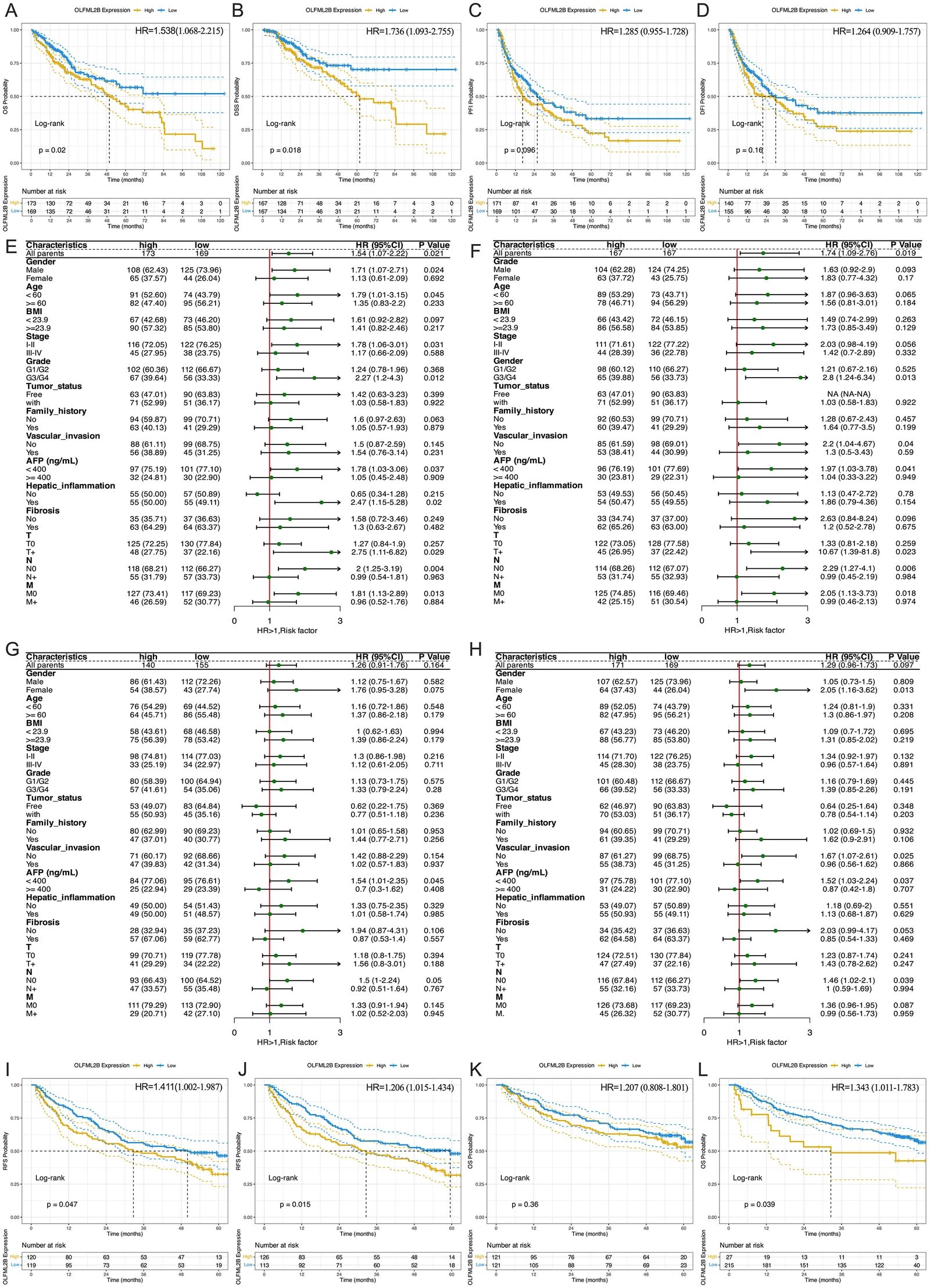
Prognostic significance of OLFML2B in patients with HCC. **(A–D)** OS **(A)**, DSS **(B)**, PFI **(C)**, and DFI **(D)** of patients with HCC stratified by OLFML2B expression in the TCGA cohort. **(E–G)** Cox regression survival analysis stratified by OLFML2B expression in the TCGA-LIHC Cohort: OS **(E)**, DSS **(F)**, PFI **(G)**, DFI **(H)**. **(I, J)** RFS analysis of HCC patients in the GSE14520 cohort stratified by median **(I)** or optimal cutoff **(J)** value of OLFML2B expression. **(K, L)** OS analysis of HCC patients in the GSE14520 cohort stratified by median **(K)** or optimal cutoff value **(L)** of OLFML2B expression. OS, overall survival; DSS, disease-specific survival; PFI, progression-free interval; DFI, disease-free interval; RFS, relapse-free survival.

Based on the TCGA-LIHC cohort, univariate Cox regression analyses demonstrated that high OLFML2B expression (defined by median grouping) served as a significant risk factor for overall survival (OS, hazard ratio (HR) = 1.538, 95% CI: 1.068–2.215, p = 0.021) and disease-specific survival (DSS, HR = 1.736, 95% CI: 1.093–2.755, p = 0.019), but no significant associations were observed with disease-free interval (DFI, HR = 1.264, 95% CI: 0.909–1.757, p = 0.164) or progression-free interval (PFI, HR = 1.285, 95% CI: 0.955–1.728, p = 0.097). Correspondingly, Kaplan-Meier curves confirmed that patients with high OLFML2B expression exhibited poorer OS (p = 0.02) and DSS (p = 0.018), with no statistically significant differences in DFI (p = 0.16) or PFI (p = 0.096) (**Figures 2A–D**). To evaluate the long-term prognostic value of OLFML2B independent of early post-treatment events, we performed landmark analyses with follow-up initiated at 3 months for PFI and 6 months for DFI. The 3-month landmark for PFI aligns with the first routine radiographic assessment after initiation of locoregional or systemic therapy, as recommended by post-treatment surveillance protocols (first imaging evaluation at 3 months after systemic therapy or radiation-based locoregional therapy, and every 3 months thereafter for the first 2 years) (20, 21). The 6-month landmark for DFI corresponds to the cutoff for “very early recurrence” following curative-intent resection or ablation, a timepoint associated with distinct pathogenesis and significantly worse prognosis (22–24). Notably, in this stratified analysis, high OLFML2B expression emerged as a significant predictor of poorer PFI (p = 0.037) and DFI (p = 0.036) (**Supplementary Figure 2**). These findings collectively indicate that OLFML2B expression is not a marker of early disease recurrence or progression; instead, it correlates with an elevated risk of late, long-term recurrence and progression specifically in patients who remain event-free during the initial post-treatment period.

Furthermore, to investigate heterogeneous prognostic impacts of elevated OLFML2B expression across diverse clinical subgroups, stratified Cox regression analyses were performed. Subgroup analyses revealed that high OLFML2B expression predicted worse OS in subgroups of male patients, those aged ≤60 years, with stage I-II disease, grade G3/G4 tumors, low alpha-fetoprotein (AFP) levels (<400 ng/mL), hepatic inflammation, or T+/N0/M0 status (**Figure 2E**). For DSS, adverse prognostic effects were observed in subgroups without vascular invasion, with low AFP, or with T+/N0/M0 status (**Figure 2F**). With respect to DFI and PFI, significant prognostic associations were restricted to the low AFP or N0 subgroups (**Figures 2G, H**). Subsequent stratified Kaplan–Meier survival curves further validated these subgroup-specific prognostic discrepancies (**Supplementary Figure 3**). Notably, high OLFML2B expression correlated with poorer OS, DSS, DFI, and PFI in low-AFP HCC patients, but no such associations were observed in high-AFP patients (**Supplementary Figures 3I, O, Q**).

To validate these prognostic associations, we utilized the GSE14520 and ICGC-LIRI-JP datasets as validation cohorts. We confirmed that high OLFML2B expression in the GSE14520 dataset is a risk factor for OS (HR = 1.73, 95% CI: 1.04–2.89, p = 0.034) and relapse-free survival (RFS, HR = 1.72, 95% CI: 1.12–2.62, p = 0.013). Subsequent stratified analyses revealed that significant differences in RFS existed between the OLFML2B high-expression and low-expression groups regardless of grouping methods (**Figures 2I, J**, median grouping: p = 0.021; optimal cut-off grouping: p = 0.015); whereas significant inter-group differences in OS were only observed under the optimal cut-off grouping strategy (**Figures 2K, L**, p = 0.36 and 0.039). In the ICGC-LIRI-JP cohort, stratification by the optimal cut-off value further supported the adverse prognostic impact of high OLFML2B expression, showing markedly worse OS in the high-expression group (**Supplementary Figure 4**).

### 3.3 OLFML2B is associated with the global immune cell landscape in HCC

**Figure 3:**
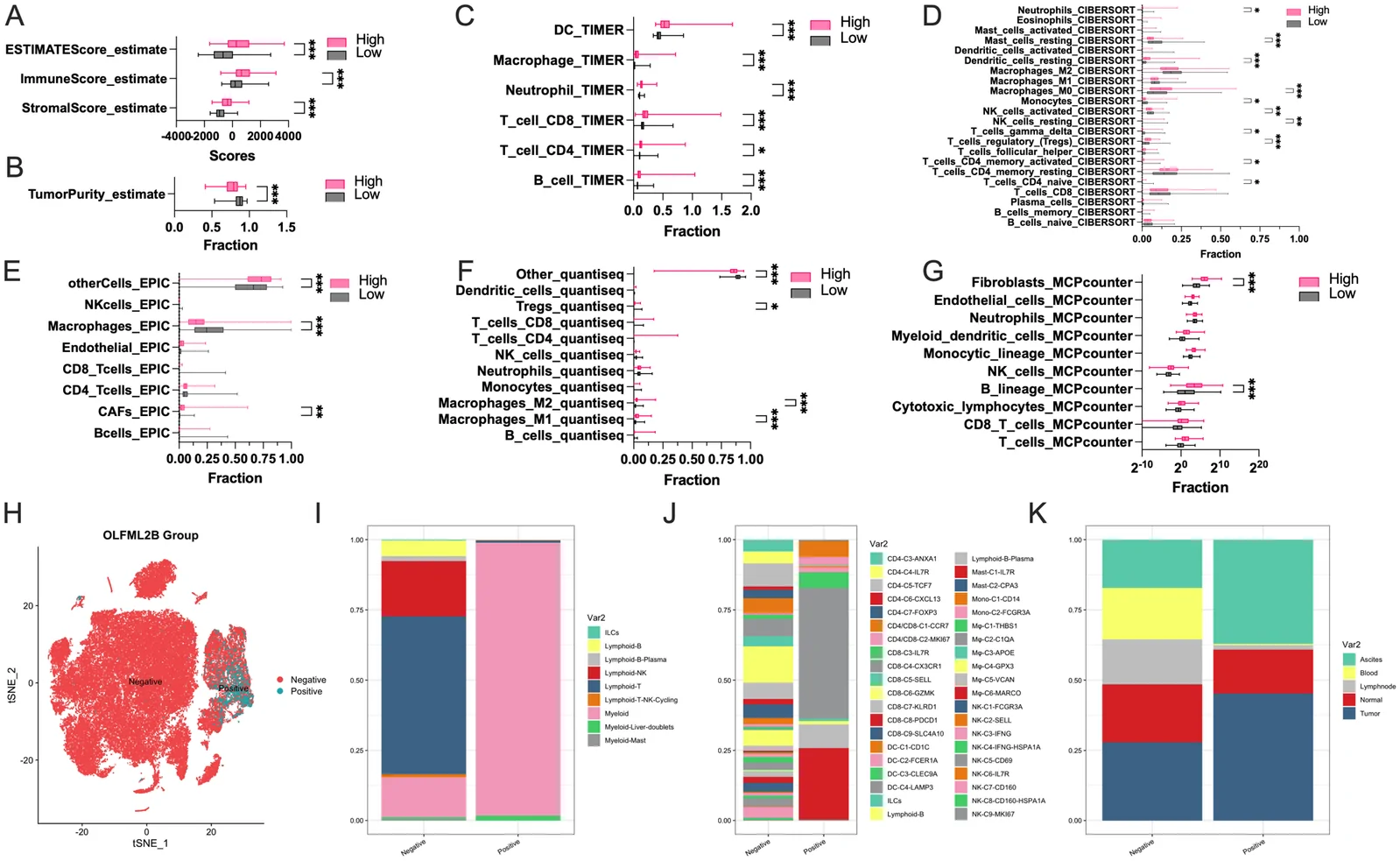
OLFML2B expression is associated with an immunosuppressive tumor microenvironment in HCC. **(A, B)** Immune score, stromal score, ESTIMATE score **(A)**, and tumor purity **(B)** were calculated by the ESTIMATE algorithm in HCC tissues with high and low OLFML2B expression. **(C–F)** Immune infiltration analysis via the TIMER **(C)**, CIBERSORT **(D)**, EPIC **(E)**, QuanTIseq **(F)**, and MCPcounter **(G)** Algorithm in OLFML2B high- and low-expression tissues from the TCGA cohort. **(H–K)** Single-cell RNA sequencing (scRNA-seq) analysis using the GSE140228 dataset. OLFML2B-Positive/Negative cell subgrouping **(H)**, global immune cell composition of OLFML2B-Positive and -Negative cells **(I)**, fine analysis of immune cell composition in OLFML2B-Positive and -Negative cells **(J)**, origin of OLFML2B-Positive and -Negative Cells **(K)**. *p< 0.05, **p< 0.01, ***p< 0.001.

To evaluate the impact of OLFML2B on the tumor immune microenvironment (TIME) in HCC, we stratified patients in the TCGA-LIHC cohort into high- and low-expression groups based on OLFML2B transcript levels for comprehensive analyses of immune infiltration. The high-OLFML2B expression group exhibited significantly elevated ESTIMATE, immune, and stromal scores, along with reduced tumor purity (**Figures 3A, B**), suggesting a more complex and immune-rich tumor microenvironment. TIMER algorithm analysis revealed that the high-expression group had higher estimated infiltration scores for dendritic cells (DCs), macrophages, neutrophils, CD8^+^ T cells, CD4^+^ T cells, and B cells (**Figure 3C**). Multiple deconvolution algorithms corroborated this pattern. CIBERSORT analysis demonstrated increased infiltration of M0, M1, and M2 macrophages, neutrophils, DCs, and multiple T cell subsets, including CD8^+^ T cells, CD4^+^ T cells, and regulatory T cells (Tregs) (**Figure 3D**). EPIC analysis indicated higher infiltration of macrophages and cancer-associated fibroblasts (CAFs) (**Figure 3E**). QuanTIseq showed increases in Tregs, and M1 and M2 macrophages (**Figure 3F**). MCPcounter analysis further confirmed elevated levels of fibroblasts and B lineage cells (**Figure 3G**). Taken together, these bulk transcriptomic analyses indicate that high OLFML2B expression is associated with an immunosuppressive, fibroblast-rich TIME, characterized by enhanced macrophage infiltration.

To gain deeper insights into the cellular specificity and tissue context of these changes, we performed single-cell RNA sequencing (scRNA-seq) analysis using the GSE140228 dataset (**Figure 3H**). This revealed three key features of the immune landscape associated with OLFML2B expression. First, OLFML2B expression was closely associated with the global composition of immune cells. Compared to the OLFML2B-negative group, the OLFML2B-positive group showed a substantial increase in the proportion of myeloid cells, accompanied by a significant decrease in lymphoid cells (including T, NK, and B cells), with cytotoxic T cells and NK cells being nearly absent (**Figure 3I**). Second, OLFML2B expression was associated with a skewed macrophage polarization. It specifically correlated with the accumulation of “pro-tumor” M2-like macrophage subsets (Mφ-2-C1QA, Mφ-3-APOE, Mφ-6-MARCO), while the proportions of “anti-tumor” M1-like macrophages (Mφ-1-THBS1) and the immunostimulatory cDC1 subset (DC-C1-CD1C) were extremely low (**Figure 3J**). Third, this association was highly specific to the HCC tumor microenvironment. Cells in the OLFML2B-positive group were predominantly derived from tumor tissues and ascites (a metastasis-related niche), whereas contributions from normal tissues, lymphoid tissues, and blood were markedly reduced (**Figure 3K**). To further validate the association between OLFML2B expression and the HCC immune microenvironment, we analyzed HCC samples from three independent single-cell transcriptome datasets (GSE125449, GSE149614, and GSE156625). In three independent GEO datasets, high OLFML2B expression consistently correlated with enhanced CAFs infiltration. Beyond CAFs, myeloid cells (in GSE149614 and GSE156625 datasets) and tumor-associated macrophages (in GSE125449 dataset) displayed a similar positive association with OLFML2B levels (**Supplementary Figure 5**).

In summary, by integrating bulk and single-cell transcriptomic analyses, we demonstrate that high OLFML2B expression is associated with an immunosuppressive and fibroblast-rich TIME in HCC. This is characterized by a myeloid-skewed infiltration landscape, preferential accumulation of pro-tumor M2 macrophages and CAFs, and the exclusion of cytotoxic lymphocytes, within a tumor-specific context.

### 3.4 Knockdown of OLFML2B suppresses the proliferation of HCC cells

**Figure 4:**
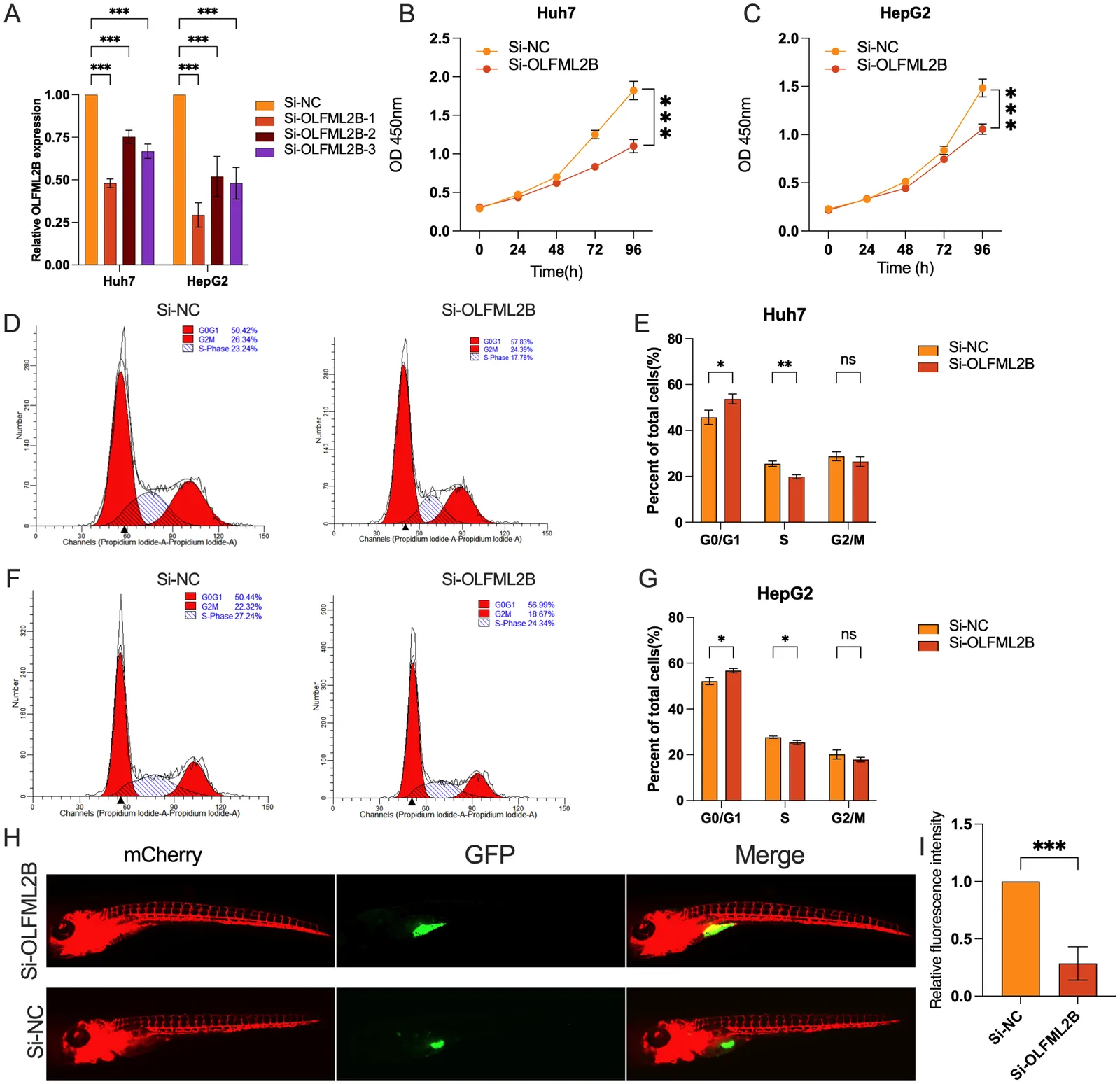
OLFML2B knockdown inhibits proliferation and cell cycle progression in HCC cells. **(A)** qPCR validation of OLFML2B knockdown in negative control (NC) and OLFML2B-knockdown (KD) Huh7 and HepG2 cells. **(B, C)** CCK-8 proliferation curves of Huh7 **(B)** and HepG2 **(C)** cells in NC and KD groups. **(D, E)** Flow cytometry cell-cycle analysis in Huh7 cells: representative histograms **(D)** and quantification of cell distribution in G1, S, and G2/M phases **(E)**. **(F, G)** Flow cytometry cell-cycle analysis in HepG2 cells: representative histograms **(F)** and quantification of phase distribution **(G)**. **(H, I)** Zebrafish xenograft tumor model. Representative fluorescence images of tumors formed by NC or KD HepG2 cells **(H)** and quantification of relative tumor proliferation **(I)**. *p < 0.05, **p < 0.01, ***p < 0.001 versus NC group.

To investigate the role of OLFML2B in HCC cell proliferation, we knocked down OLFML2B expression in Huh7 and HepG2 cell lines using specific siRNAs. The transfection efficiency was confirmed by RT-qPCR (**Figure 4A**). Subsequent functional assays revealed that OLFML2B knockdown significantly inhibited the proliferation of both cell lines, as measured by the CCK-8 assay (**Figures 4B, C**). Cell cycle analysis further demonstrated that OLFML2B silencing led to an accumulation of cells in the G0/G1 phase and a concomitant reduction in the S and G2/M phases in both Huh7 and HepG2 cells (**Figures 4D–G**), suggesting a blockade of cell cycle progression. Comparable effects were observed upon OLFML2B knockdown in Hep3B cells (**Supplementary Figure 6A**).

To validate these findings in an *in vivo* context, we employed a zebrafish xenograft model. GFP-labeled control or stable OLFML2B-knockdown HepG2 cells were microinjected into 48 hpf zebrafish embryos. Consistently, at 2 days post-injection (dpi), the fluorescent area and intensity, indicative of tumor burden, were significantly lower in the OLFML2B-knockdown group compared to the control group (**Figures 4H, I**). Taken together, the *in vitro* and *in vivo* findings demonstrate that OLFML2B knockdown suppresses HCC cell proliferation by inducing G0/G1 cell cycle arrest.

### 3.5 Knockdown of OLFML2B inhibits the migration and invasion of HCC cells

**Figure 5:**
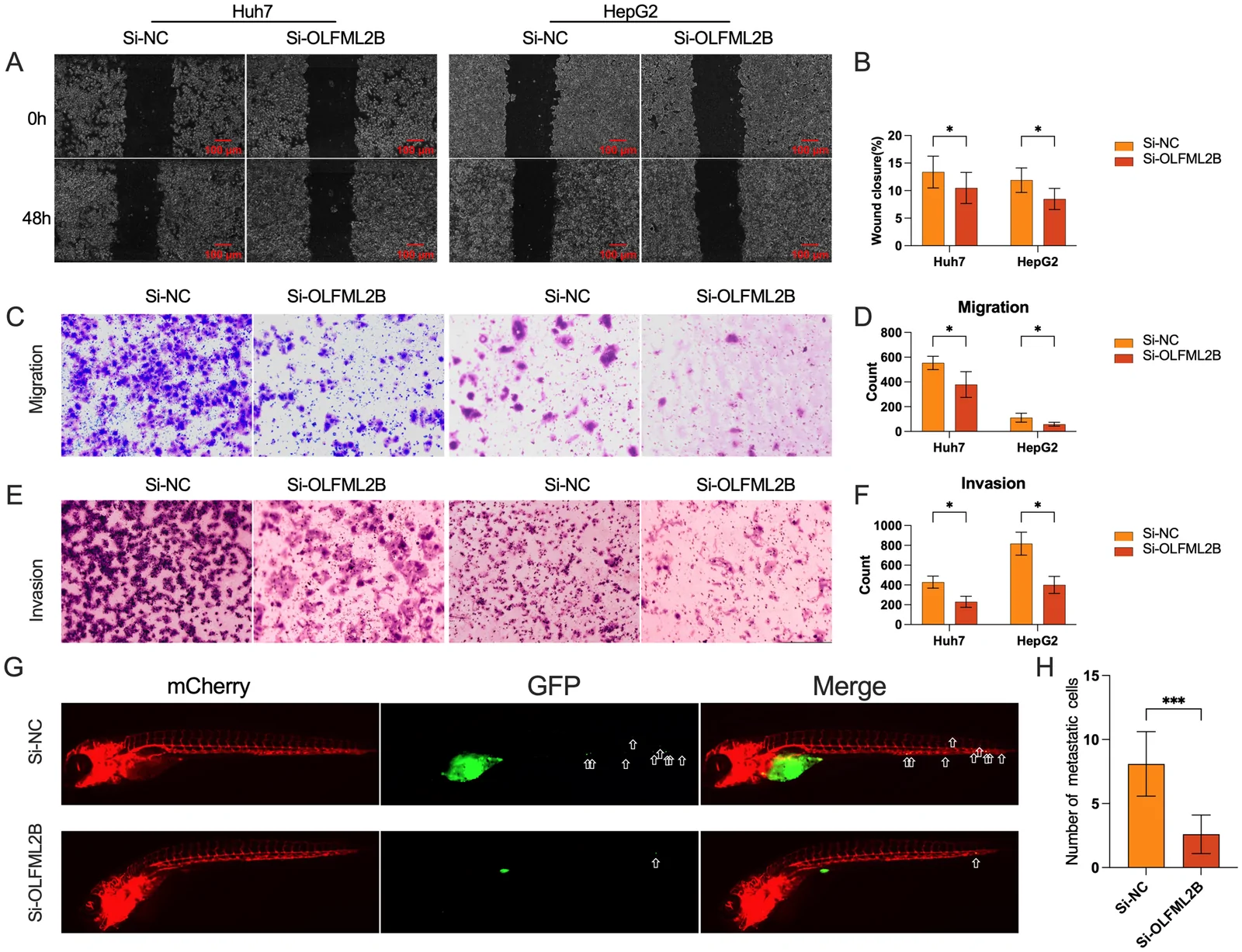
Inhibition of HCC cell metastasis by OLFML2B knockdown *in vitro* and *in vivo*. **(A)** Representative scratch-wound images at 0 and 48 h in NC and KD Huh7 and HepG2 cells. **(B)** Quantification of wound-closure rate at 48 h. **(C, D)** Transwell migration assay. Representative images **(C)** and quantification of migrated cells **(D)** in NC and KD groups. **(E, F)** Matrigel-based Transwell invasion assay. Representative images **(E)** and quantification of invaded cells **(F)** in NC and KD groups. **(G, H)** Zebrafish xenograft metastasis assay. Representative fluorescence images of disseminated HepG2 cells **(G)** and quantification of metastatic cells **(H)**. *p < 0.05, ***p < 0.001 versus NC group.

Wound healing assays revealed that OLFML2B knockdown significantly reduced wound closure rates in Huh7, HepG2, and Hep3B cells compared to negative controls (**Figures 5A, B**; **Supplementary Figures 6B, C**). Moreover, OLFML2B knockdown in these cell lines markedly decreased the number of migrating and invasive cells (**Figures 5C–F**; **Supplementary Figures 6D–F**). Additionally, in a zebrafish xenotransplantation model, HepG2 cells with stable OLFML2B knockdown exhibited significantly reduced metastatic capability compared to negative control cells (**Figures 5G, H**). Together, these findings indicate that knockdown of OLFML2B in HCC cells substantially suppresses their metastatic potential.

### 3.6 Knockdown of OLFML2B promotes apoptosis in HCC cells

**Figure 6:**
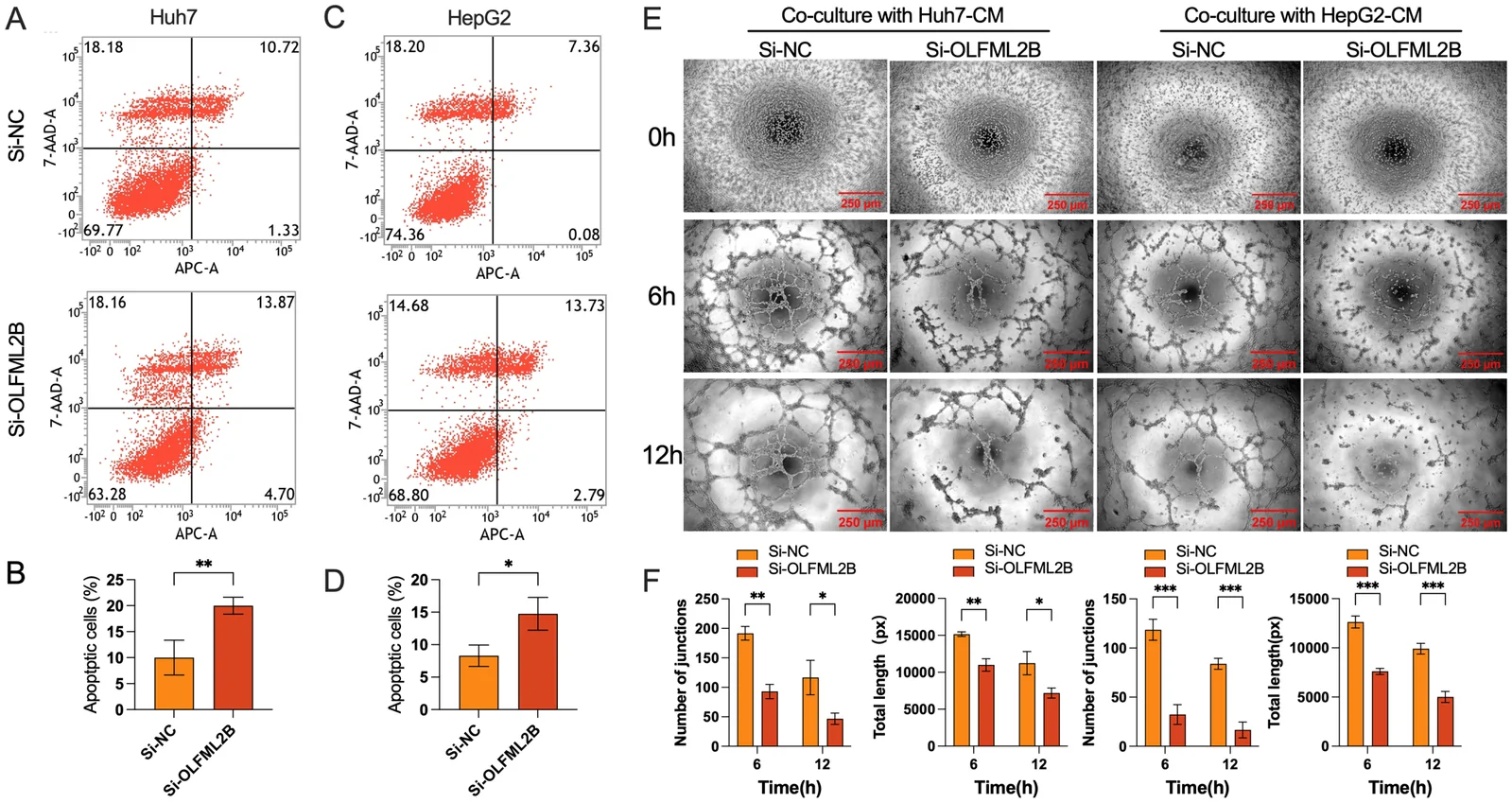
OLFML2B knockdown promotes apoptosis and inhibits angiogenesis in HCC cells. **(A, B)** Flow cytometry analysis of apoptosis in Huh7 cells. Representative 7-AAD/PI staining plots **(A)** and quantification of apoptotic cell percentage **(B)** in NC and KD cells. **(C, D)** Flow cytometry analysis of apoptosis in HepG2 cells. Representative 7-AAD/PI staining plots **(C)** and quantification of apoptotic cell percentage **(D)** in NC and KD cells. **(E, F)** HUVEC tubule-formation assay. Representative images **(E)** and quantification of total tubule length and number of junctions **(F)** after culture with conditioned medium from NC or KD HepG2 cells. CM, conditioned medium. *p < 0.05, **p < 0.01, ***p < 0.001.

To assess the role of OLFML2B in regulating apoptosis, we performed 7-AAD/PI staining and flow cytometry analysis in OLFML2B-knockdown Huh7 and HepG2 cells. The results showed that OLFML2B knockdown significantly increased the total apoptotic rate (early + late apoptosis) in both cell lines compared to the negative controls (**Figures 6A–D**).

### 3.7 Knockdown of OLFML2B in HCC cells inhibits angiogenesis *in vitro*

In a tubule formation assay, culture supernatants from OLFML2B-knockdown Huh7 and HepG2 cells significantly reduced the number of tubule junctions and the total tubule length in HUVECs compared with supernatants from negative control cells (**Figures 6E, F**). This result suggests that OLFML2B depletion in HCC cells impairs their ability to promote angiogenesis *in vitro*.

### 3.8 OLFML2B knockdown inhibits EMT via PI3K/AKT pathway

**Figure 7:**
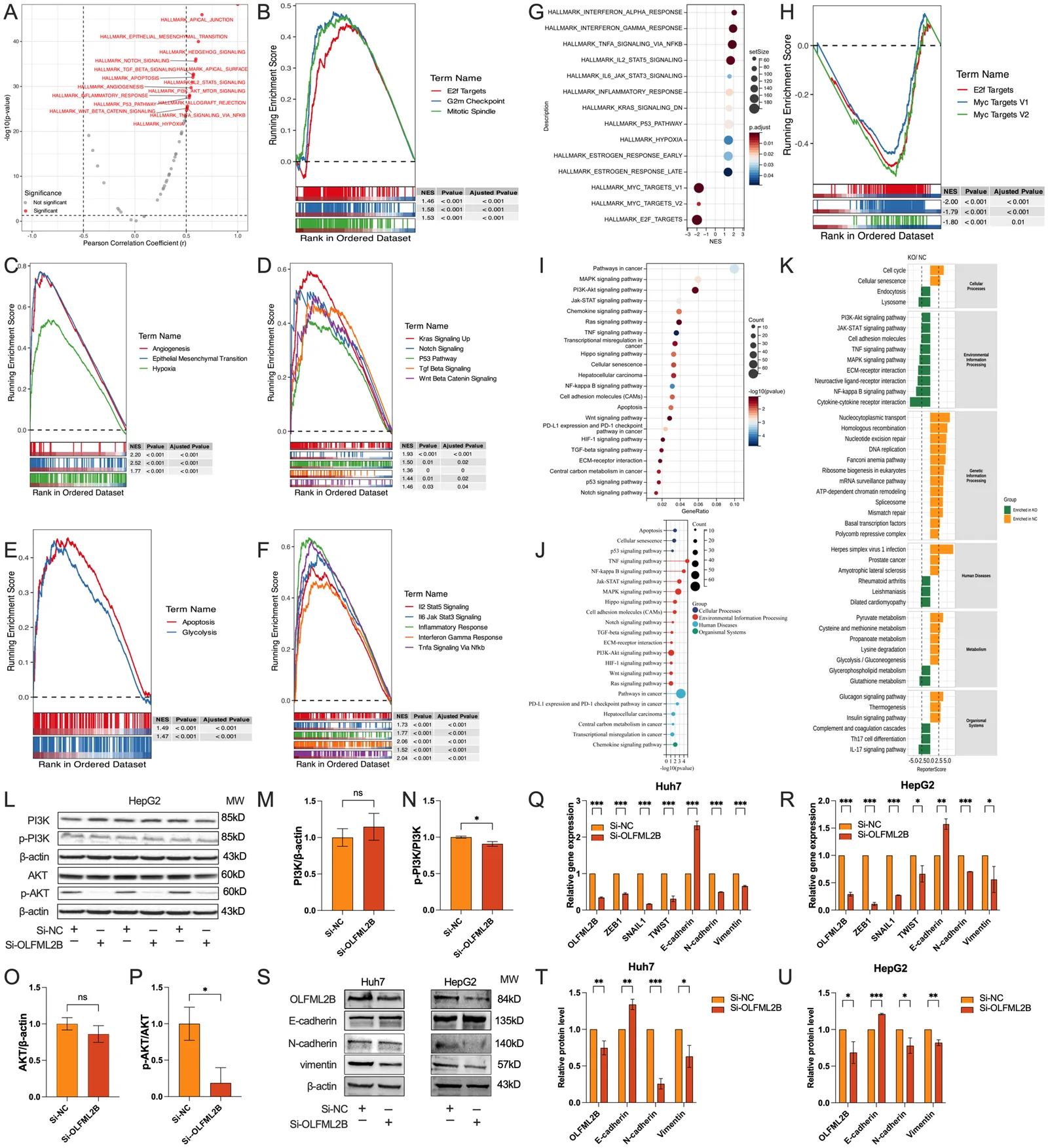
OLFML2B knockdown suppresses EMT in HCC cells by inhibiting the PI3K/AKT pathway. **(A)** GSVA volcano plot of hallmark gene sets that are significantly correlated with OLFML2B expression in the TCGA-LIHC cohort (FDR < 0.05). (B-F) GSEA enrichment plots of hallmark gene sets enriched in high- vs. low-OLFML2B expression groups (TCGA-LIHC): proliferation-related **(B)**, angiogenesis/EMT/hypoxia **(C)**, key signaling pathway **(D)**, apoptosis/glycolysis **(E)**, and immune-related **(F)**. **(G)** GSEA identifies hallmark gene sets enriched in KD versus NC HepG2 cells. **(H)** GSEA plot of proliferation-related gene sets enriched in NC versus KD HepG2 cells. **(I, J)** Bubble plot **(I)** and bar plot **(J)** of KEGG pathways enriched from DEGs (|Log_2_FC| > 0.584, p < 0.1) in OLFML2B-KD vs. NC HepG2 cells. **(K)** GSEA of KEGG gene sets enriched in KD vs. NC HepG2 cells. **(L)** Western blot of PI3K/AKT pathway proteins and their phosphorylation in NC and KD HepG2 cells. (M-P) Quantification of PI3K/β-actin **(M)**, p-PI3K/PI3K **(N)**, AKT/β-actin **(O)**, and p-AKT/AKT **(P)** ratios. **(Q, R)** RT-qPCR analysis of EMT-related marker expression in NC and KD Huh7 **(Q)** and HepG2 **(R)** cells. **(S)** Western blot of EMT-related proteins in NC and KD Huh7 and HepG2 cells. **(T, U)** Quantification of N-cadherin, Vimentin, and E-cadherin protein levels in NC and KD Huh7 **(T)** and HepG2 **(U)** cells. *p < 0.05, **p < 0.01, ***p < 0.001.

To comprehensively characterize OLFML2B-related biological processes and signaling pathways in HCC, we integrated GSVA and GSEA analyses, revealing its involvement in critical oncogenic processes driving HCC. Specifically, OLFML2B was enriched in pathways regulating cell proliferation and cell cycle (PI3K/Akt-mTOR, WNT-β-catenin, hedgehog, Notch, E2F Targets, G2/M Checkpoint, Mitotic Spindle), apoptosis and tumor suppression (apoptosis, p53), tumor metastasis and microenvironment remodeling (EMT, angiogenesis, TGF-β signaling), immune and inflammatory responses (inflammatory response, TNF-α/NF-κB, IL2-STAT5, IL6-JAK-STAT3), and metabolic adaptation and stress response (hypoxia, glycolysis) (**Figures 7A–F**).

To validate these findings, we constructed a stable OLFML2B knockdown (KD) cell line (validated by Western blotting, **Supplementary Figures 7**, **8**) and performed transcriptome sequencing followed by GSEA, KEGG, and GRSA. GSEA showed that HCC proliferation-related Hallmark gene sets (E2F Targets, Myc Targets V1/V2) were significantly downregulated in KD cells vs. controls (NC) (NES = -2.01, -1.79, -1.81, P < 0.001) (**Figures 7G, H**), indicating that OLFML2B depletion inhibits cell-cycle and proliferation pathways. Differential expression analysis identified 826 upregulated genes and 346 downregulated genes (to incorporate a broader set of potential differentially expressed genes, we set the screening criterion as |Log2FC| > 0.584, p < 0.1) compared to NC cells (**Supplementary Figure 7**), which were then subjected to KEGG pathway enrichment analysis. The results revealed that these differentially expressed genes (DEGs) were significantly enriched in pathways including Pathways in cancer, MAPK, PI3K/AKT, and Jak-STAT signaling pathways (**Figures 7I, J**). GRSA further confirmed enrichment of pathways linked to HCC cellular processes (cell cycle, senescence), environmental information processing (PI3K/AKT, JAK-STAT, MAPK, NF-κB), and immune function (Toll-like receptor, IL-17 signaling) in KD cells (**Figure 7K**). Collectively, these results suggest that OLFML2B may play a critical role in HCC progression by regulating cell proliferation, immune microenvironment, and metabolic processes.

The PI3K/AKT signaling pathway is critically involved in driving tumor cell proliferation, invasion, and malignant progression. To investigate whether OLFML2B is involved in the PI3K/AKT signaling pathway, we detected the protein levels of key pathway components. Compared with the NC group, there were no significant differences in the total protein levels of PI3K and AKT; however, the phosphorylation levels of PI3K (p-PI3K) and AKT (p-AKT) were significantly reduced (**Figures 7L–P**; **Supplementary Figures 9**, **10**). These findings indicate that OLFML2B knockdown in HepG2 cells inhibits PI3K/AKT signaling. EMT serves as a pivotal process in tumor progression, enabling cancer cells to acquire invasive, metastatic, and drug-resistant phenotypes. The PI3K signaling cascade is critically involved in regulating tumor EMT progression and modulating the expression of core EMT markers (e.g., E-cadherin, N-cadherin), thereby driving malignant transformation. RT-qPCR revealed that E-cadherin expression was significantly increased in OLFML2B-knockdown Huh7 and HepG2 cells, while the expression levels of N-cadherin, vimentin, ZEB1, SNAIL1, and TWIST decreased (**Figures 7Q, R**). Western blot analysis further confirmed that OLFML2B knockdown resulted in a marked increase in E-cadherin and a reduction in N-cadherin and vimentin (**Figures 7S–U**; **Supplementary Figure 11**). These results suggest that OLFML2B might activate the EMT pathway in HCC cells.

To further verify that OLFML2B promotes HCC progression through the PI3K/AKT signaling pathway, we performed rescue experiments using the PI3K-specific activator 740Y-P. OLFML2B knockdown significantly suppressed cell migration and invasion in Huh7 and HepG2 cells, as demonstrated by wound healing and Transwell assays (**Supplementary Figure 12**); however, these inhibitory effects were largely reversed upon co-treatment with 740Y-P (**Supplementary Figure 12**). Western blot analysis further revealed that OLFML2B silencing markedly reduced AKT phosphorylation, accompanied by downregulation of N-cadherin and vimentin and upregulation of E-cadherin (**Supplementary Figures 13**, **14**), indicative of suppressed EMT. Notably, the PI3K activator restored p-AKT levels and partially rescued the expression of these EMT biomarkers in OLFML2B-silenced cells (**Supplementary Figures 13**; **14**). Collectively, these findings suggest that OLFML2B exerts its oncogenic functions, at least in part, by activating the PI3K/AKT axis and inducing EMT.

### 3.9 OLFML2B knockdown enhances HCC cell sensitivity to targeted drugs

EMT typically reduces tumor cell sensitivity to chemotherapeutics and targeted agents by enhancing anti-apoptotic capacity and stemness. To evaluate the sensitizing effect of OLFML2B knockdown (KD) on multi-kinase inhibitors of HCC cells, the OLFML2B knockdown (KD) cells were treated with a concentration gradient of Sorafenib, Cabozantinib, and Regorafenib. The half maximal inhibitory concentration (IC_50_) curves were generated at 24 and 48 hours post-treatment, and drug interactions were quantified by the Bliss independence method and the Chou–Talalay combination index (CI). OLFML2B silencing enhanced the cytotoxicity of sorafenib, regorafenib, and cabozantinib in both HepG2 and Huh7 cells; however, the magnitude of sensitization differed considerably by cell line, drug, and time point.

**Figure 8:**
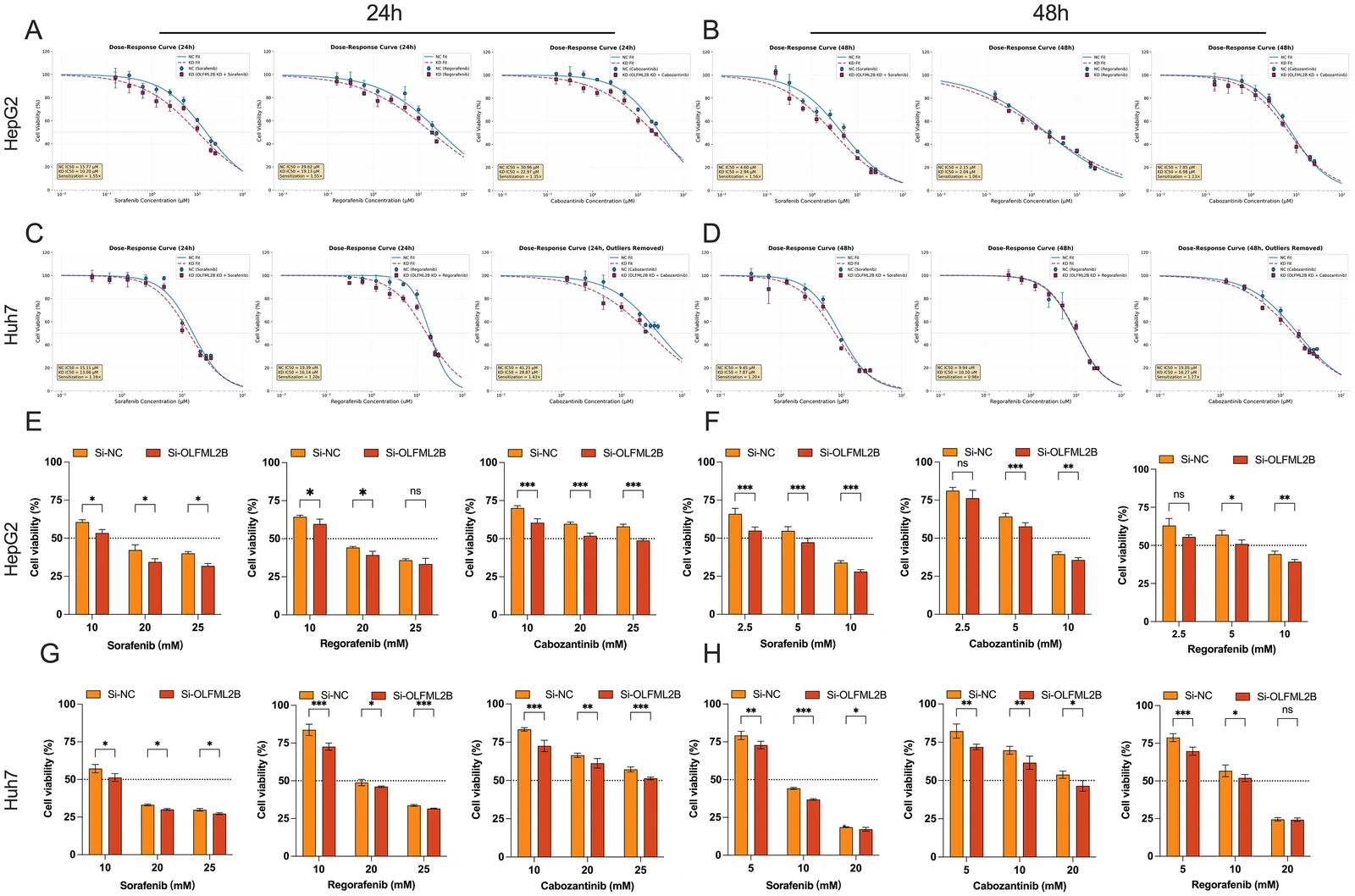
OLFML2B knockdown sensitizes HCC cells to targeted therapeutics. **(A, B)** Dose-response curves of NC and KD HepG2 cells treated with sorafenib, regorafenib, or cabozantinib for 24 h **(A)** and 48 h **(B)**. **(C, D)** Cell viability of NC and KD Huh7 cells under increasing concentrations of sorafenib, regorafenib, and cabozantinib for 24 h **(C)** and 48 h **(D)**. **(E, F)** Cell viability of NC and KD HepG2 cells treated with sorafenib, regorafenib, or cabozantinib for 24 h **(E)** and 48 h **(F)**. **(G, H)** Cell viability of NC and KD Huh7 cells under increasing concentrations of sorafenib, regorafenib, and cabozantinib for 24 h **(G)** and 48 h **(H)**. *p < 0.05, **p < 0.01, ***p < 0.001 versus NC group.

In HepG2 cells, OLFML2B knockdown resulted in robust, stable sensitization to sorafenib. At 24 h, the IC_50_ of sorafenib decreased from 15.78 μM (95% CI: 13.53–18.39) to 10.20 μM (95% CI: 7.98–13.05), corresponding to a 35.3% reduction and a 1.55-fold sensitization. This effect persisted at 48 h, with IC_50_ values of 4.60 μM (NC) versus 2.94 μM (KD), yielding a 1.56-fold sensitization (36.1% reduction) (**Figures 8A, B**). Bliss analysis identified synergistic interactions (excess > 5%) across the 0.625–25 μM range at 24 h and the 0.312–10 μM range at 48 h, with CI geometric means of 0.486 and 0.583, respectively, indicating overall synergy at both time points (**Supplementary Figures 15A, B**). By contrast, sensitization to regorafenib was markedly time-limited. At 24 h, OLFML2B KD reduced the regorafenib IC_50_ from 29.62 μM to 19.13 μM (35.4% reduction, 1.55-fold sensitization), with a CI geometric mean of 0.485 and Bliss excess values exceeding 5% across 0.625–25 μM. However, after 48 h, the curves nearly overlapped (IC_50_: 2.15 μM vs. 2.04 μM), the sensitization ratio decreased to 1.05-fold (5.1% reduction), and the CI geometric mean increased to 0.951, indicating an overall additive effect (**Figures 8A, B**; **Supplementary Figures 15A, B**). Similarly, cabozantinib sensitization was prominent at 24 h (IC_50_: 30.96 μM vs. 22.97 μM; 25.8% reduction, 1.35-fold; CI = 0.372) but attenuated at 48 h (IC_50_: 7.85 μM vs. 6.98 μM; 11.1% reduction, 1.12-fold; CI = 0.659), with Bliss excess declining from >10% at 5–10 μM to only 5.0% at 2.5 μM (**Figures 8A, B**; **Supplementary Figures 15A, B**).

In Huh7 cells, the sensitization patterns diverged. For sorafenib, OLFML2B KD produced modest but consistent sensitization: 1.16-fold at 24 h (IC_50_: 15.11 μM vs. 13.06 μM) and 1.20-fold at 48 h (IC_50_: 9.45 μM vs. 7.87 μM) (**Figures 8C, D**). Bliss analysis revealed a concentration-dependent expansion of the synergistic window from 5–10 μM at 24 h to 2.5–10 μM at 48 h, with CI geometric means of 0.646 and 0.650, respectively (**Supplementary Figures 15C, D**). Regorafenib exhibited a striking time-dependent loss of synergy: at 24 h, KD reduced the IC_50_ from 19.39 μM to 16.14 μM (16.7% reduction, 1.20-fold), with pronounced synergy in the 5–10 μM range (Bliss excess 15.5–16.8%) and a CI geometric mean of 0.372; at 48 h, the IC_50_ values converged (9.94 μM vs. 10.10 μM), Bliss excess fell within ±1%, and the CI geometric mean reached 0.903, indicating additivity (**Figures 8C, D**; **Supplementary Figures 15C, D**). Cabozantinib followed a comparable trajectory, with strong synergy at 24 h (IC_50_: 41.21 μM vs. 28.87 μM; 29.9% reduction, 1.43-fold; CI = 0.631; Bliss excess up to 10.5% at 20 μM) that weakened substantially by 48 h (IC_50_: 19.05 μM vs. 16.27 μM; 14.6% reduction, 1.17-fold; CI = 0.875), where all concentrations yielded additive effects (**Figures 8C, D**; **Supplementary Figures 15C, D**). Additionally, compared with the NC group, cell viability was significantly decreased in the OLFML2B knockdown group treated with three concentrations (10, 20, and 25 μM) for 24 h and 48 h (**Figures 8E–H**). Collectively, OLFML2B knockdown tends to sensitize HCC cells to sorafenib, regorafenib, and cabozantinib predominantly at 24 h, with this sensitizing potency weakening markedly at 48 h. These observations suggest that OLFML2B influences the drug responsiveness of HCC cells to these targeted agents.

## 4 Discussion

Hepatocellular carcinoma (HCC) remains a major global health burden with high mortality, largely due to limited understanding of its underlying molecular mechanisms and a lack of effective prognostic biomarkers and therapeutic targets. This study systematically explored the role of OLFML2B in HCC to reveal its clinical significance and regulatory mechanisms, offering novel insights into HCC pathogenesis and treatment.

Abnormally high OLFML2B expression in HCC tissues is a key molecular feature with significant diagnostic value. By integrating 17 HCC-related datasets from TCGA, ICGC, and GEO, this study found that OLFML2B expression in HCC tissues was significantly higher than in non-tumor, cirrhotic, and dysplastic liver tissues. Moreover, this high expression is not restricted to etiology, with stable upregulation observed in both HBV- and NASH-associated HCC, suggesting that OLFML2B is a core molecular event in HCC development rather than a secondary response to inflammation or fibrosis. ROC analysis of 15 datasets confirmed its excellent diagnostic efficacy, with all AUC values exceeding 0.7 and 3 datasets exceeding 0.9. This conclusion aligns with the study by Wang et al., which demonstrated OLFML2B’s differential high expression in HCC and its strong positive correlation with LINC01116 (correlation coefficient = 0.810); combining these two genes with other markers further improved HCC diagnostic accuracy (AUC ≥ 0.700), providing a new potential tool for solving the clinical challenge of early HCC diagnosis (25). While similar high-expression patterns have been reported in other tumors like gastric cancer and ovarian cancer (11, 26), its core value lies in addressing unmet needs in HCC diagnosis. Collectively, these findings indicate that high OLFML2B expression is a potential prognostic marker for unfavorable outcomes in patients with HCC.

In terms of prognosis assessment, OLFML2B has critical predictive significance for HCC patient survival. This study showed that HCC patients with high OLFML2B expression had significantly poorer overall survival (OS) and disease-specific survival (DSS), and this correlation was more pronounced in subgroups, including male patients, those with stage I-II disease, and those with low AFP levels. The prognostic relevance of OLFML2B is consistent with prior work by Liu et al., who identified its high expression as an independent predictor of unfavorable outcomes via multivariate Cox regression (HR = 1.268, 95% CI: 1.076–1.495) after incorporating OLFML2B into an HCC prognostic gene signature (27). While its prognostic value has also been demonstrated in osteosarcoma (serving as a core gene in a prognostic model with AUC > 0.997 in internal validation and 0.832–0.929 in external cohorts) and triple-negative breast cancer (TNBC) (16, 28), its utility in stratifying high-risk HCC patients, especially the clinically challenging low-AFP subgroup, remains its most notable clinical significance.

The regulatory effect of OLFML2B on HCC malignant phenotypes is the core mechanism of its involvement in disease progression. *In vitro* experiments confirmed that silencing OLFML2B significantly inhibited the proliferation of HepG2 and Huh7 cells, induced G0/G1 cell cycle arrest, reduced cell migration, invasion, and angiogenesis, and promoted HCC cell apoptosis. In the zebrafish xenograft model, HCC cells with OLFML2B knockdown exhibited a markedly reduced proliferation rate accompanied by impaired metastatic capacity, confirming its tumor-promoting role in HCC growth and metastasis. This role is common across other tumors. Ge et al. found that OLFML2B can promote the proliferation, colony formation, and invasion of clear cell renal cell carcinoma (ccRCC) cells, and Loh et al. pointed out that it is persistently highly expressed in circulating tumor cells (CTCs) of neuroblastoma and associated with disease recurrence (13, 28).

Additionally, OLFML2B expression was associated with an immunosuppressive tumor microenvironment (TME) in HCC. Transcriptomic and single-cell analyses (TIMER, CIBERSORT, and scRNA-seq) revealed that high OLFML2B levels correlated with increased infiltration of pro-tumor immune cells, including M2 macrophages, cancer-associated fibroblasts (CAFs), and regulatory T cells (Tregs), as well as decreased abundance of anti-tumor immune cells such as cytotoxic T cells and NK cells. This is consistent with Wang et al.’s findings that OLFML2B is closely related to HCC immune infiltration, showing a negative correlation with tumor purity and a positive correlation with specific immune cell types (25); Wu et al. also found a similar correlation between OLFML2B and M2 macrophage infiltration in TNBC (16). Nevertheless, as these findings are derived from bioinformatic association analyses, the causal relationship and underlying mechanisms by which OLFML2B may regulate the tumor immune microenvironment remain elusive. Direct functional evidence from *in vitro* and *in vivo* studies is warranted to clarify whether OLFML2B actively shapes immune cell infiltration and function in HCC.

Mechanistically, this study demonstrated that OLFML2B may promote HCC progression, at least in part, through PI3K/AKT-EMT signaling. GSEA, GRSA, and Western blot analyses showed that silencing OLFML2B can inhibit the PI3K/AKT signaling pathway, downregulate mesenchymal markers (N-cadherin, vimentin) and EMT transcription factors (ZEB1, SNAIL1), and upregulate the epithelial marker E-cadherin, thereby suppressing HCC cell invasion and metastasis. Liu et al. also verified OLFML2B’s involvement in PI3K/Akt pathway regulation and HCC malignant progression (11). Similar findings have been reported in mechanistic studies of OLFML2B in other tumors. For example, Hu et al. confirmed that it is regulated by the SNHG10/hsa-miR-378a-3p ceRNA axis in gastric cancer (29), and Ren et al. found that it cooperates with the PI3K/Akt pathway to regulate tumor progression in gastric cancer (30). These studies provide cross-cancer references for analyzing the molecular mechanism of OLFML2B.

OLFML2B also has potential therapeutic relevance in HCC. This study found that silencing OLFML2B enhanced HCC cell sensitivity to targeted drugs such as sorafenib, cabozantinib, and regorafenib, reducing IC50 values by 11%–35%, suggesting that OLFML2B is a key influencing factor in HCC-targeted therapy resistance. Given that targeted drug resistance is the main cause of HCC treatment failure, interventions targeting OLFML2B, such as combining its inhibition with existing targeted drugs, may offer a new strategy to improve efficacy. Yin et al. noted that high-OLFML2B-expressing high-risk osteosarcoma is more sensitive to targeted therapies like the Hsp90 inhibitor 17-AAG (31), further supporting OLFML2B’s potential as a therapeutic target. At the same time, Wang et al.’s report of a synergistic regulatory relationship between OLFML2B and LINC01116 provides a direction for exploring combined treatment strategies for HCC (25).

Although this study revealed the significance of OLFML2B in HCC, several limitations should be acknowledged. First, *in vitro* experiments relied on HepG2 and Huh7 cell lines without validation in primary HCC cells, and the observed IC_50_ reduction was modest (11–35%) with drug testing restricted to merely two cell lines; besides, OLFML2B overexpression validation in drug-resistant cell lines to confirm causal links was not performed in the current work. Second, while zebrafish xenograft models are widely used to monitor tumor progression and metastatic potential, they cannot fully recapitulate the intricate tumor microenvironment of human HCC due to deficiencies in the adaptive immune system and authentic human stromal interactions. Third, the specific molecular targets linking OLFML2B to PI3K/AKT-EMT and immune modulation remain undefined; Fourth, the majority of our clinical findings are derived from retrospective analyses, necessitating prospective validation in independent cohorts. Moving forward, future studies should: (i) validate findings in diverse clinical cohorts and primary cell models; (ii) Construct orthotopic tumor models (e.g., mouse orthotopic models) to mimic the endogenous liver microenvironment faithfully; (iii) identify direct OLFML2B interactors and dissect the underlying mechanistic axis; (iv) establish OLFML2B-overexpressing drug-resistant HCC cell lines to clarify its causal role in drug sensitivity; and (v) advance targeted therapeutic strategies to facilitate the clinical translation of OLFML2B.

In summary, OLFML2B is upregulated in HCC and contributes to malignant phenotypes, including cell proliferation, migration, and invasion, through the PI3K/AKT-EMT axis. Its expression correlates with an immunosuppressive tumor microenvironment enriched in pro-tumor immune cells. These findings suggest that OLFML2B may serve as a diagnostic and prognostic biomarker, providing a rationale for exploring targeted therapeutic strategies in HCC.

## Data Availability

The datasets presented in this study can be found in online repositories. Specifically, the transcriptomic sequencing data generated in this study are available from the GEO database under accession GSE315394. The repository names and accession numbers for the remaining datasets are listed in the article/**Supplementary Material**.

## Supplementary material

Supplementary Figure 1 OLFML2B protein expression is upregulated in HCC tissues. Representative immunohistochemical (IHC) staining of OLFML2B in paired HCC and adjacent non-tumor tissues from six patients. Stronger brown cytoplasmic staining indicates higher OLFML2B protein levels in HCC tissues compared to adjacent non-tumor tissues. Scale bars: 200 μm (40×) and 50 μm (100×).

Supplementary Figure 2 Landmark survival analysis of HCC patients stratified by OLFML2B expression. **(A, B)** Kaplan-Meier curves for progression-free interval (PFI) **(A)** and disease-free interval (DFI) **(B)** with follow-up initiated at landmark times of 3 months and 6 months, respectively, comparing high vs. low OLFML2B expression groups.

Supplementary Figure 3 Prognostic impact of OLFML2B expression across clinical and pathological subgroups in HCC. **(A-I)** Kaplan-Meier overall survival (OS) curves stratified by high vs. low OLFML2B expression in patients grouped by gender **(A)**, age **(B)**, tumor stage **(C)**, tumor grade **(D)**, hepatitis status **(E)**, T stage **(F)**, N stage **(G)**, M stage **(H)**, and AFP level **(I)**. **(J-N)** Disease-specific survival (DSS) curves in patients grouped by AFP level **(J)**, T stage **(K)**, N stage **(L)**, M stage **(M)**, and tumor stage **(N)**. **(O, P)** Disease-free interval (DFI) curves in patients grouped by AFP level **(O)** and N stage **(P)**. **(Q, R)** Progression-free interval (PFI) curves in patients grouped by AFP level **(Q)** and N stage **(R)**. Data are presented as Kaplan-Meier estimates; the log-rank test was used to compare the high-expression groups.

Supplementary Figure 4 Prognostic significance of OLFML2B in patients with HCC in the ICGC-LIRI-JP cohort. (A, B) OS analysis of HCC patients in the ICGC-LIRI-JP cohort stratified by median **(A)** or optimal cutoff value **(B)** of OLFML2B expression: OS, overall survival.

Supplementary Figure 5 Single-cell RNA sequencing (scRNA-seq) analysis using the GSE125449, GSE149614, and GSE156625 datasets.

Supplementary Figure 6 OLFML2B knockdown suppresses the proliferation and metastatic potential of Hep3B cells. **(A)** CCK-8 proliferation curves of Huh7 **(B)** and HepG2 **(C)** cells in NC and KD groups. **(B)** Representative scratch-wound images at 0 and 48 h in NC and KD Hep3B cells. **(C)** Quantification of wound-closure rate at 48 h. **(D–F)** Transwell migration assay. Representative images **(D)** and quantification of migrated cells **(E)** and invaded cells **(F)** in NC and KD groups.

Supplementary Figure 7 OLFML2B knockdown efficiency and transcriptomic profiling in HepG2 cells. **(A)** Western blot analysis confirming OLFML2B knockdown (KD) at the protein level in stable HepG2 cell lines. **(B)** Volcano plot depicting differentially expressed genes (DEGs) identified by RNA sequencing between negative control (NC) and OLFML2B-knockdown (KD) HepG2 cells (KD vs. NC). **(C)** Heatmap of the top 50 genes significantly dysregulated by RNA sequencing in control and OLFML2B-knockdown HepG2 cells (KD vs. NC). Genes were filtered using the thresholds of |log_2_FC| > 0.584 and p < 0.1.

Supplementary Figure 8 Western blot analysis confirming OLFML2B knockdown (KD) at the protein level in stable HepG2 cell lines. Si-NC: HepG2 transfect with PLKO.1-NC-shRNA plasmid; Si-OLFML2B-1 and Si-OLFML2B: HepG2 transfect with PLKO.1-OLFML2B-shRNA-1 and PLKO.1-OLFML2B-shRNA-2 plasmid, respectively.

Supplementary Figure 9 Western blotting was performed to determine the expression levels of PI3K and its phosphorylated form (p-PI3K). **(A)** The expression levels of PI3K were determined by Western blotting and normalized to β-actin; suffixes -1, -2, and -3 indicate three replicate experiments. **(B)** The expression levels of p-PI3K were determined by Western blotting and normalized to PI3K; suffixes -1, -2, and -3 indicate three replicate experiments.

Supplementary Figure 10 Western blotting was performed to determine the expression levels of AKT and its phosphorylated form (p-AKT). **(A)** The expression levels of AKT were determined by Western blotting and normalized to β-actin; suffixes -1, -2, and -3 indicate three replicate experiments. **(B)** The expression levels of p-AKT were determined by Western blotting and normalized to AKT; suffixes -1, -2, and -3 indicate three replicate experiments.

Supplementary Figure 11 Western blotting was performed to determine the expression levels of EMT markers. **(A)** The expression of OLFML2B in Huh7 cells transfected with PLKO.1-OLFML2B-shRNA-1, -2, -3, and -4, respectively, was determined by Western blotting and normalized to β-actin, Si-NC (PLKO.1-NC-shRNA) as a negative control. **(B–D)** The expression of E-cadherin **(B)**, N-cadherin **(C)**, Vimentin **(D)**, and β-actin **(E)** in Huh7 cells transfected with PLKO.1-OLFML2B-shRNA-1, -2, -3, and -4, respectively, was determined by Western blotting and normalized to β-actin, Si-NC (PLKO.1-NC-shRNA) as a negative control. **(F)** The expression of OLFML2B in HepG2 cells transfected with different PLKO.1-OLFML2B-shRNA was determined by Western blotting and normalized to β-actin, Si-NC (PLKO.1-NC-shRNA) as a negative control. **(G–J)** The expression of E-cadherin **(G)**, N-cadherin **(H)**, Vimentin **(I)**, and β-actin **(J)** in HepG2 cells transfected with different PLKO.1-OLFML2B-shRNA was determined by Western blotting and normalized to β-actin, Si-NC (PLKO.1-NC-shRNA) as a negative control—suffixes -1, -2, -3 indicating PLKO.1-OLFML2B-shRNA-1, -2, -3, respectively.

Supplementary Figure 12 Rescue experiments validating the role of OLFML2B in HCC cell migration and invasion. Huh7 and HepG2 cells were subjected to OLFML2B knockdown (si-OLFML2B) or control (si-NC) with or without 740-YP treatment. **(A–C)** Wound healing assays; **(D–H)** Transwell migration and invasion assays. Data are mean ± SD (n = 3). *P < 0.05, **P < 0.01, ***P < 0.001, ****P < 0.0001.

Supplementary Figure 13 740-YP partially restores PI3K/AKT activation and EMT marker expression in OLFML2B-knockdown HCC cells. Huh7 and HepG2 cells were transfected with si-NC or si-OLFML2B and subsequently treated with DMSO or 740-YP (10 μM). (A) Representative Western blot images of total AKT and phosphorylated AKT (p-AKT, Ser473). (B) Western blot analysis of EMT-related proteins, including E-cadherin, N-cadherin, and vimentin.

Supplementary Figure 14 Western blot analysis of PI3K/AKT signaling and EMT markers in rescue experiments.

Supplementary Figure 15 Synergy analysis of OLFML2B knockdown combined with sorafenib, regorafenib, or cabozantinib in HepG2 and Huh7 cells. Cells were transfected with si-NC or si-OLFML2B and treated with the indicated multi-kinase inhibitors (TKIs) at serial concentrations for 24 h or 48 h. Drug interactions were evaluated by the Chou–Talalay median-effect principle and the Bliss independence model. **(A)** HepG2 cells, 24 h treatment. **(B)** HepG2 cells, 48 h treatment. **(C)** Huh7 cells, 24 h treatment. **(D)** Huh7 cells, 48 h treatment. For each cell line and time point, the left panels show median-effect plots (log(fa/fu) vs. log(concentration)) with linear regression fits for NC (blue) and KD (red) groups; the middle panels show Bliss independence analysis (Bliss excess, %) with synergy threshold set at +5% (green, synergy; yellow, additive; red, antagonism); and the right panels show Chou–Talalay combination index (CI) plots with synergy (CI < 0.9), additive (0.9 ≤ CI ≤ 1.1), and antagonism (CI > 1.1) thresholds. Data points represent the mean of six replicates. NC, negative control; KD, OLFML2B knockdown; fa, fraction affected; fu, fraction unaffected.

## Glossary

AFP: Alpha-fetoprotein
AUC: area under the curve
AVR-CC: active viral replication chronic carriers
CAFs: cancer-associated fibroblasts
CC: chronic carriers
CCK-8: Cell counting kit-8
ccRCC: clear cell renal cell carcinoma
CI: Confidence interval
CTCs: circulating tumor cells
DCs: dendritic cells
DEGs: differentially expressed genes
dpi: days post-injection
DSS: disease-specific survival
EMT: epithelial-mesenchymal transition
FBS: Fetal bovine serum
FDR: False Discovery Rate
GEO: Gene Expression Omnibus
GFP: green fluorescent protein
GRSA: Generalized Range Swap Algorithm
GSEA: Gene Set Enrichment Analysis
HBV: Hepatitis B virus
HCC: Hepatocellular carcinoma
HGD/LGD: high-grade/low-grade dysplastic
hpf: hours post-fertilization
HR: Hazard ratio
HUVEC: human umbilical vein endothelial cells
IC: half maximal inhibitory concentration
ICGC: International Cancer Genome Consortium
KD: knockdown
MS-222: tricaine methane sulfonate
NASH: non-alcoholic steatohepatitis
NC: Negative control
NES: Normalized Enrichment Score
NIH: National Institutes of Health
OLF: olfactomedin
OS: overall survival
PBS: phosphate-buffered saline
PI: propidium iodide
PTU: 1-phenyl-2-thiourea
PVDF: polyvinylidene difluoride
qRT-PCR: Real-Time Quantitative Reverse Transcription PCR
RFS: relapse-free survival
ROC: receiver operating characteristic
scRNA-seq: single-cell RNA sequencing
SDS-PAGE: sodium dodecyl sulfate-polyacrylamide gel electrophoresis
STR: short tandem repeat
TCGA: The Cancer Genome Atlas Program
TIME: tumor immune microenvironment
TME: tumor microenvironment
TNBC: triple-negative breast cancer
Tregs: regulatory T cells
