# Supplementary material for: OLFML2B promotes hepatocellular carcinoma malignancy via the PI3K/AKT-EMT axis and correlates with an immunosuppressive tumor microenvironment
Source: Front Immunol. 2026 Aug 10;17:1779625. doi: 10.3389/fimmu.2026.1779625 (PMC13531038; doi:10.3389/fimmu.2026.1779625)
Supplement: Supplementary file 1 [file Table1.docx]

Table S1 Detail information of datasets used in this study

| No. | Dataset | Platform | Type | Tissues | Reference |
| --- | --- | --- | --- | --- | --- |
| 1 | GSE22058 | GPL6793 | Expression profiling by array | 192 samples (96 resected tumor and 96 adjacent non-tumor liver tissues) | PMID:20739924  PMID:22403344 |
| 2 | GSE25097 | GPL10687 | Expression profiling by array | 268 HCC tumor, 243 adjacent non-tumor, 40 cirrhotic and 6 healthy liver samples | PMID:21955977 |
| 3 | GSE36376 | GPL10558 | Expression profiling by array | 240 tumor and 193 non-tumor liver tissues | PMID:23800896 |
| 4 | GSE14520 | GPL571  GPL3921 | Expression profiling by array | Cohort1：22 tumor-normal pairs of HCC samples; Cohort2: 42 tumor-normal pairs of HCC samples | PMID:21159642 |
| 5 | GSE54236 | GPL6480 | Expression profiling by array | 77 adjacent nontumorous samples and 78 HCC samples. | PMID:25666192 |
| 6 | GSE63898 | GPL13667 | Expression profiling by array | 228 HCC and 168 cirrhotic samples | PMID:25645722 |
| 7 | GSE64041 | GPL6244 | Expression profiling by array | 60 tumor-normal pairs HCC samples, and 5 normal liver samples | PMID:27499918 |
| 8 | GSE76427 | GPL10558 | Expression profiling by array | 115 primary tumors (PTs) and 52 adjacent non-tumor tissues (ANTTs) derived from 115 HCC patients. | PMID:29117471 |
| 9 | GSE60502 | GPL96 | Expression profiling by array | 18 HCC and paired adjacent non-tumorous liver tissue | PMID:25376302 |
| 10 | GSE102079 | GPL570 | Expression profiling by array | 152 tumorous and 91 adjacent liver tissues from HCC patients as well as 14 normal liver | PMID:29454748 |
| 11 | GSE164760 | GPL13667 | Expression profiling by array | 53 NASH-HCCs, 29 adjacent non-tumor NASH liver samples, 74 NASH samples,6 healthy liver and 8 cirrhotic liver samples. | PMID:33992698 |
| 12 | GSE6764 | GPL570 | Expression profiling by array | 7 advanced HCC; 13 cirrhotic liver tissue; 10 early HCC; 7 high-grade dysplastic liver tissue; 10 low-grade dysplastic liver tissue; 10 normal liver tissue; 10 very advanced HCC; 8 very early HCC | PMID:17393520 |
| 13 | GSE135631 | GPL16791 | Expression profiling by high throughput sequencing | 15 tumor-normal pairs of HBV associated HCC samples |  |
| 14 | GSE202853 | GPL11154  GPL21290 | Expression profiling by high throughput sequencing | 10 tumor-normal pairs of HCC samples | PMID:35657344  PMID:38830975 |
| 15 | GSE46444 | GPL13369 | Expression profiling by array | 83 HCC and 47 liver cirrhosis samples | PMID:24498002 |
| 16 | TCGA-LIHC |  | Expression profiling by high throughput sequencing | 373 HCC and 50 adjacent non-tumor liver tissues, among which 365 patients with overall survival time longer than 30 days. |  |
| 17 | ICGC-LIRI-JP |  | Expression profiling by high throughput sequencing | 243 HCC and 202 adjacent non-tumorous liver tissues, including 198 tumor-normal pairs of HCC patients |  |
| 18 | GSE140228 | GPL20301 | Single-cell transcriptome profiling | 11 blood samples, 11 liver tissues, 7 primary tumor tissues, 3 tumor edge tissues, 3 tumor core tissues, 4 ascites samples and 3 lymph node tissues were collected from HCC patients. | PMID:31675496 |
| 19 | GSE125449 | GPL18573  GPL20301 | Single-cell transcriptome profiling | Set 1: 7 HCC and 2 intrahepatic cholangiocarcinoma (iCCA) samples  Set 2: 2 HCC and 5 iCCA samples | PMID:31588021 |
| 20 | GSE149614 | GPL24676 | Single-cell transcriptome profiling | 10 HCC primary tumors (PT), 8 adjacent non-tumor livers (NTL), 2 portal vein tumor thrombi (PVTT), and 1 metastatic lymph node (MLN) | PMID:35933472  PMID:38479553 |
| 21 | GSE156625 | GPL16791  GPL17021 | Single-cell transcriptome profiling | 57 HCC samples, 1 healthy normal liver sample, 2 specimens for each gestational stage of human fetal liver (14-, 16-, 18- and 21-week), as well as 12 mouse liver samples | PMID:32976798 |

Table S2 Sequences of the primers used in this study

| Name | Forward 5' to 3') | | Reverse (5' to 3') |
| --- | --- | --- | --- |
| OLFML2B | | ACTGCTTCGTCATCTGTGGG | GGGACGATCTGTGTGTTGGT |
| β-actin | | CTACCTCATGAAGATCCTCACCGA | TTCTCCTTAATGTCACGCACGATT |
| ZEB1 | | TGAATGGGCGACCAAGAACA | ACTGCCTGGTGATGCTGAAA |
| SNAIL | | TCCACAAGCACCAAGAGTCC | AAAGTCCTGTGGGGCTGATG |
| TWIST1 | | GCTGATTGGCACGACCTCT | CACCATCCTCACACCTCTGC |
| E-cadherin | | GGGGTCTGTCATGGAAGGTG | GAAACTCTCTCGGTCCAGCC |
| N-cadherin | | ATCAACCCCATACACCAGCC | ACTAACCCGTCGTTGCTGTT |
| Vimentin | | GCTGATTGGCACGACCTCT | CACCATCCTCACACCTCTGC |

Table S3 Antibodies used blotting in this study

| Antibody | Source |
| --- | --- |
| β-actin Rabbit Polyclonal Antibody | Biosharp，China |
| Vimentin Polyclonal Antibody | CST，USA |
| N-Cadherin Polyclonal Antibody | CST，USA |
| E-Cadherin Polyclonal Antibody | CST，USA |
| OLFML2B Polyclonal Antibody | BOSTER，China |
| P-PI3K p85 (Tyr458) | CST，USA |
| PI3K | CST，USA |
| P-AKT (Ser473) | CST，USA |
| AKT | CST，USA |

| Table S5 Univariate cox regression analysis of OLFML2B expression and survival endpoints in patients with HCC (TCGA-LIHC cohort) | | | | | | |
| --- | --- | --- | --- | --- | --- | --- |
| Endpoints | B | SE | Wald | df | p-value | HR (95%CI) |
| OS | 0.432 | 0.186 | 5.362 | 1 | 0.021 | 1.538 (1.068-2.215) |
| DFI | 0.234 | 0.168 | 1.939 | 1 | 0.164 | 1.264 (0.909-1.757) |
| DSS | -0.551 | 0.236 | 5.470 | 1 | 0.019 | 1.736 (1.093-2.755) |
| PFI | 0.251 | 0.151 | 2.749 | 1 | 0.097 | 1.285 (0.955-1.728) |
